# Metallic sample preparation for phase transformation analysis

**DOI:** 10.1016/j.mex.2019.09.041

**Published:** 2019-10-07

**Authors:** A. Paganotti, C.V.X. Bessa, L.S. Silva, R.A.G. Silva

**Affiliations:** a Laboratory of Materials and Mechanical Manufacture, Federal University of São Paulo, Diadema, Brazil; b Maua Institute of Technology, Sao Caetano do Sul, Brazil

**Keywords:** Metallic sample preparation, Metals and alloys, Sample preparations, Heat treatment

## Abstract

Metallic materials characterization is a important part of phase transformation studies, and the sample preparation plays a crucial role in the measured properties. The methodologies available are not fully described and refer in its majority only to iron-based and aluminum-based alloys focusing in industrial analysis. In this paper the authors fully described the preparation methodology for metallic samples used by their research group. This method aims to attend different characterization techniques, producing samples of complex metallic systems from pure base materials by arc melting.

• *The melting of metallic ingots from pure base materials was fully described.*
• *The correct sample cutting, sanding and polishing procedures were described.*
• *A full detailed procedure is described to prepare the samples for different analyses.*

## Graphical abstract

**Figure:**
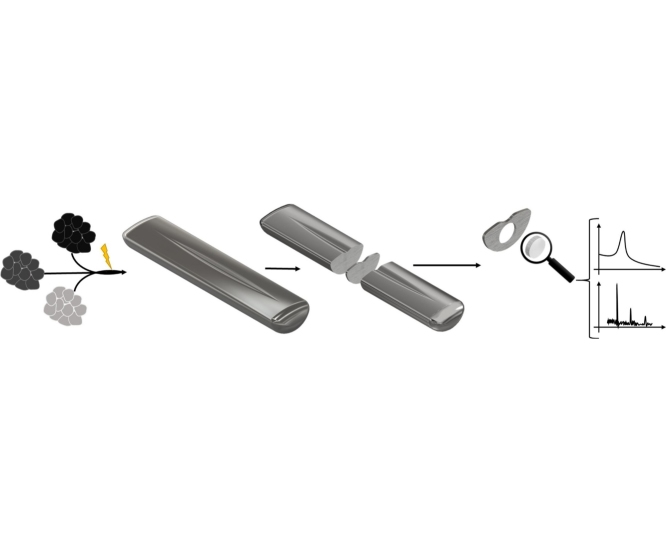

**Specification Table**

**Table:** 

Subject Area:	*Materials Science*
More specific subject area:	*Sample preparation*
Method name:	*Metallic sample preparation*
Name and reference of original method:	*Details in the background*
Resource availability:	*Not applicable*

## Background

To properly study the properties of alloys it is necessary to prepare a small ingot and then into several samples with defined characteristics. The method by which these samples are prepared influences drastically the obtained results leading to different physical properties and behaviors. In the last years, the authors have been questioned about their methodology and created this method to standardize and fully explain the preparation of metallic samples by arc melting. This method is a detailed procedure compiling the authors experience in preparing and analyzing metallic samples from alloys such as: CuAl [[1], [2], [3], [4], [5]]; SnCu [6]; CuAlZr [4]; CuAlCo [4]; CuAlSn [5]; CuAlGd [2,3,5]; CuAlMn [[1], [2], [3],^5^,[7], [8], [9], [10]]; SnCuAg [6]; CuAlAg [1]; FeNiCo [11]; CuAlMnAg [1,[7], [8], [9], [10],[12], [13], [14], [15]]; CuAlMnSn [5]; CuAlMnGd [2,3,5].

## Method details

To produce samples and measure its properties in an accurate manner three major steps are required. The first step is to produce a small ingot with the desired chemical composition. This ingot needs to be big enough so that any mass loss is negligible in comparison with its mass. On another hand, the ingot needs to be small enough so that making it from high purity metals does not turn into an expensive endeavor or produces excessive amounts of material. The second step consists into the heat treatment of the ingots or samples. This can be done using various heat treatments and different temperatures and heating rates. In the third step the sample needs to be fashioned for the desired characterization. The structure of this method will consist into describing these three processes: ingot fabrication, thermal treatments and samples preparation. It is important to notice that this method provides a detailed description of the used procedures, tips and recommendations used by this research group and by any means does not invalidates others groups procedures or recommendations.

### Ingot fabrication

**Fig. 1:**
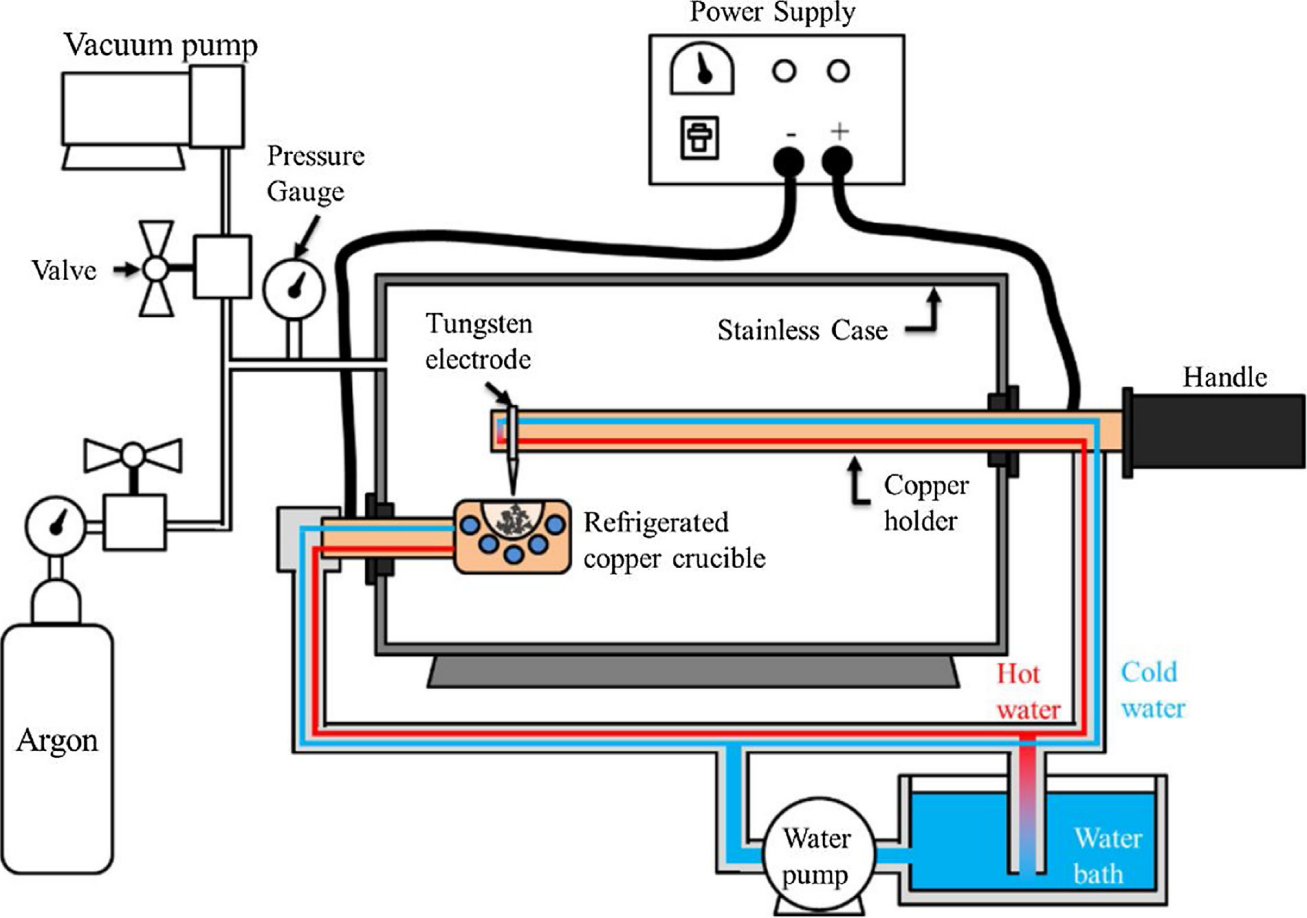
Electric arc furnace setup.

An arc melt furnace with inert atmosphere melts the base metals into an ingot, which allows a better control of the ingot composition and contaminations. In this technique, a welder power supply is connected to a non-consumable tungsten electrode and to a refrigerated crucible. The goal of this process is to produce an electric arc that passes through the base metals melting them into an alloy without melting the tungsten electrode or the crucible. The electrode, crucible and base materials are placed inside a sealed chamber enabling all the process to occur under an inert gas atmosphere, avoiding contamination. This setup is illustrated in Fig. 1.

To start the fabrication procedure the copper crucible and tungsten electrode are sanded with a 1200 grit silicon carbide sandpaper to ensure that the crucible and electrode surfaces are residues free. Then the crucible, tungsten electrode and chamber are cleaned thoroughly with practical grade acetone to remove any oils and surface contaminants. After this cleaning procedure, all the previously weighted high purity elements are placed inside the copper crucible. This placement is made so that the element with higher density stays on top and the element with the lowest density stays on the bottom of the crucible. This aims to maximize the homogenization of the ingot, as the denser element when melted tend to sink and the less dense element tends to float, easing the atoms diffusion process. After the material placement, the furnace chamber is closed to modify its atmosphere.

**Fig. 2:**
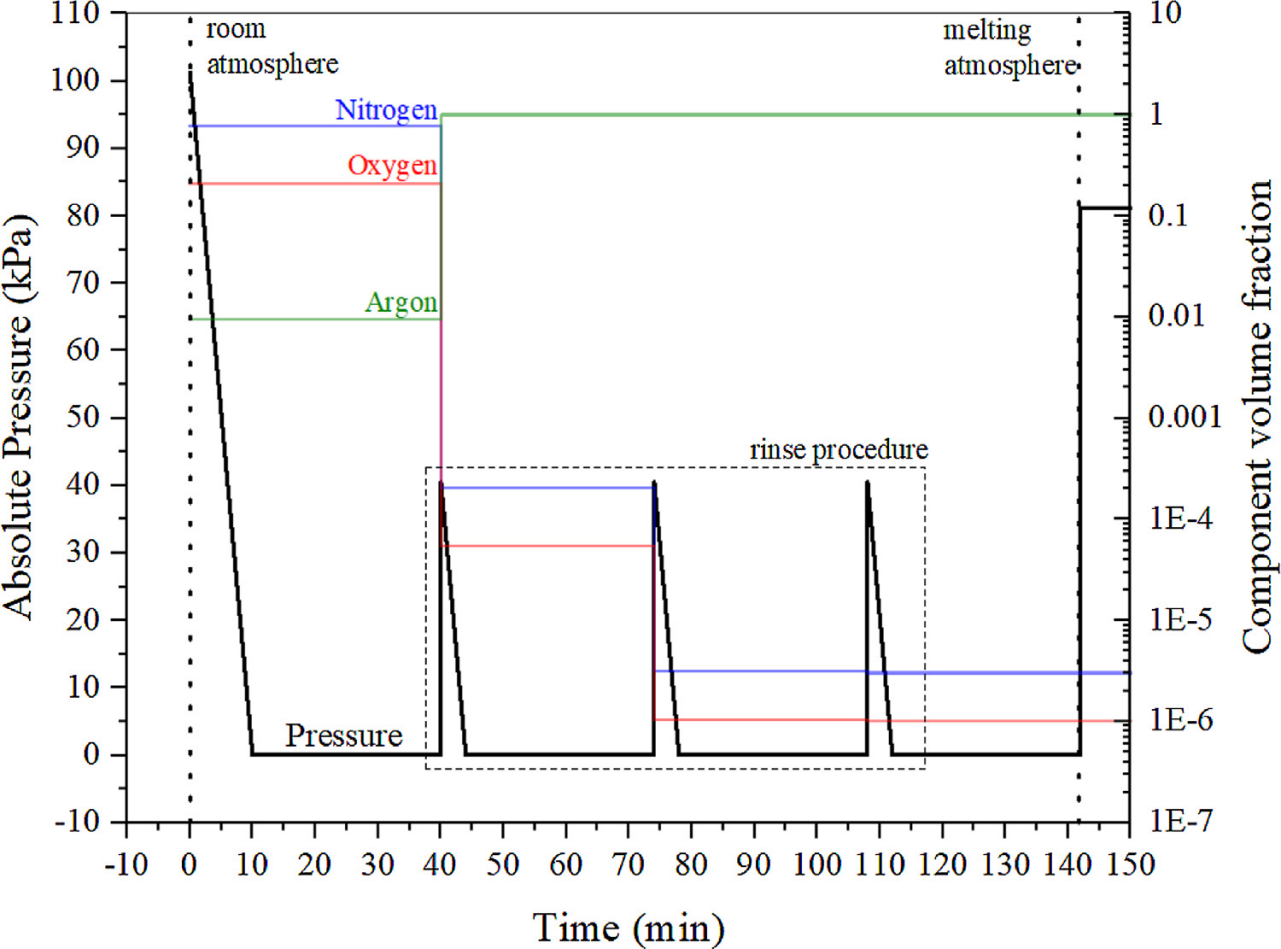
Atmosphere preparation for arc melting procedure.

As the liquid metal can reach high temperatures in this process, any molecular oxygen/nitrogen in the atmosphere can react with it, contaminating the ingot. To remove as much oxygen as possible the chamber is rinsed and pressurized with high purity Argon gas, this procedure is shown in Fig. 2. To prepare the atmosphere for the melting process a vacuum pump evacuates the chamber until the final pressure reaches around 30 Pa. The initial pressure drop is rather quick, however, to reach the desired vacuum level it is necessary to let the pump on for 30 min. After this period of time, the pump is turned off and high purity Argon gas (99.999%) pressurizes the chamber until it reaches 40 kPa. Then, the vacuum pump evacuates the furnace chamber once again. This procedure is then repeated two more times (in a total of three times) to ensure the chamber atmosphere is clean, eliminating as much as possible of the molecular oxygen. After the procedure, the chamber is pressurized with high purity argon gas until it reaches 81 kPa with O_2_ concentration at about 1 ppm. This final composition represents the atmospheric conditions during the melting procedure. The pressure of the chamber and its composition is shown in Fig. 2 along the preparation procedure.

**Fig. 3:**
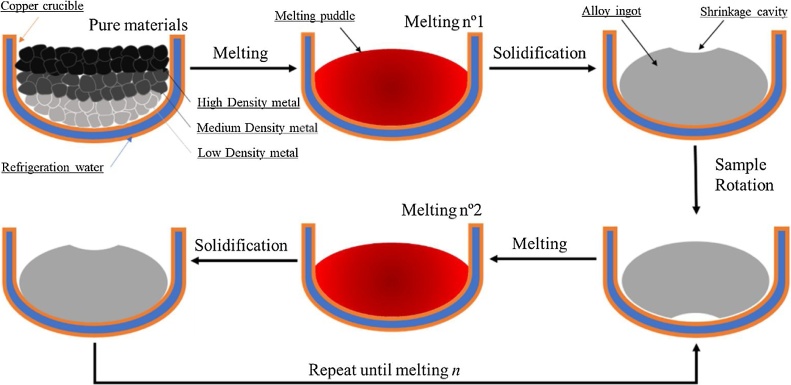
Melting and casting processes of the alloy ingot.

After the atmosphere preparation, a pump circulates water inside the crucible walls to refrigerate it. The electric arc generated between the copper crucible and the tungsten electrode melts the metals, forming a melting puddle. The puddle wets the crucible exchanging heat very quickly and solidifying. As the material solidifies the crucible acts like a refrigerated mold forming an ingot from the liquid metal. To not let the liquid metal mixture to overheat, as soon as all the material is melted, the electric arc is turned off. As the ingot is formed from pure elements this process can lead to a not homogenous ingot. To address this problem the ingot is flipped along its major axis and is then re-melted. This turning procedure is repeated *n* times to ensure the best possible homogenization of the metals. Ideally, as higher the *n*, the better homogenization will be achieved, however some alloys such high entropy ones can get very brittle after the first melting, or in case of alloys with light elements the mass loss increases rapidly. The melting procedure is illustrated in Fig. 3.

**Fig. 4:**
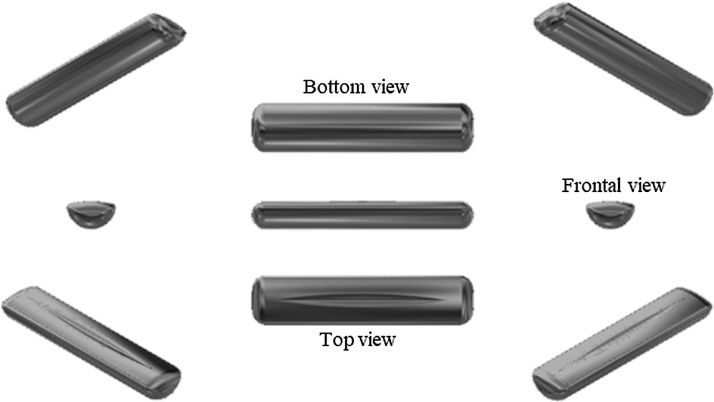
Final produced ingot.

After the final melting, the produced ingot is let to cool to room temperature inside the crucible. After this, the furnace chamber is opened to the atmosphere and the ingot is then retrieved. The final size of the ingot produced by the group depends on the total mass aimed during the procedure. However, all the produced ingot presents the shape shown in Fig. 4.

### Heat treatments

The aim of the heat treatment is to stabilize, form or decompose the desired phases of the metallic material. Basically, it can form the most stable phases by the annealing of the sample, the metastable phases during quenching and study the decomposition of these phases during heating or aging [[16], [17], [18], [19], [20], [21]].

**Fig. 5:**
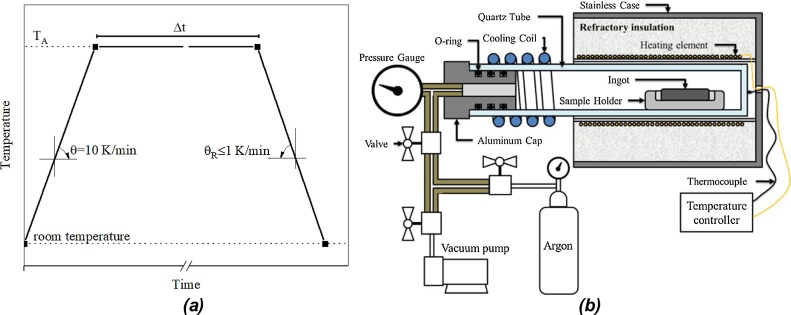
(a)Temperature vs time profile and (b) the furnace for the annealing heat treatment.

The annealing heat treatment consists of the slow cooling from a determined annealing temperature (T_A_) to produce the most stable phases from the decomposition of the phase stabilized at T_A_. If the ingot remains enough time at T_A_ this treatment also promotes the homogenization of the ingot, as enough time and energy are provided for the atomic diffusion. Three factors are important in this treatment, the homogenization time (Δt), the annealing temperature T_A_ and the cooling rate (θ_R_). The typical temperature vs time profile for the annealing treatment is shown in Fig. 5a. It is important to notice that the cooling rate must be slow enough to allow all diffusive transitions take place, typically assuming values lower than 1 K/min. As this treatment takes place in a long period of time it is necessary to perform it under an inert atmosphere. Fig. 5b shows the annealing furnace used to perform the heat treatment. The inert atmosphere is obtained in a similar procedure like that described to the arc furnace, and its composition is analogous to the melting atmosphere (shown in Fig. 2). An example performed by the group for this treatment can be seen in [22].

**Fig. 6:**
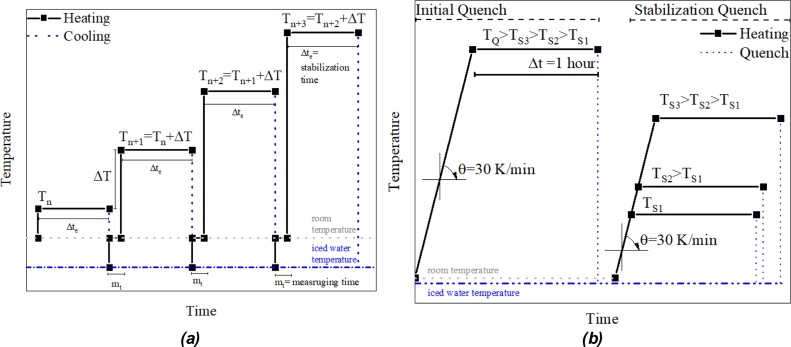
(a) Isochronal heating and (b) consecutive quenches.

After annealing, it is also necessary to study the behavior of the stable phases produced during heating. To do so it is possible to implement an isochronal heating treatment. In this treatment, the annealed sample is placed into an oven with temperature T_n_ during a time Δt_e_, subsequently, the sample is then submitted to a fast cooling into iced water to retain the stabilized phases at T_n_. The desired property is then measured and the sample is replaced into the oven at a temperature T_n+1_, which corresponds to the initial temperature (T_n_) plus an increment (ΔT) during Δt_e_. The isochronal treatment allows the study the transformation of the most stable phases at low temperatures into the high-temperature phases. Three factors are important in this treatment, the initial temperature (T_n_), the temperature increment (ΔT) and the stabilization time (Δt_e_). The typical temperature vs time profile for the isochronal heating treatment is shown in Fig. 6a. An example performed by the group for this treatment can be seen in [22].

Another treatment that provides samples to the study the transformation of formed phases is the stabilization by quenching. In this treatment, the sample is initially submitted to a quenching from a high-temperature (T_Q_). After this initial quenching, the sample is then heated up until a temperature T_S_ for a determined period of time to stabilize the phases formed from the decomposition of the metastable phases. The sample is then submitted to another quenching and then the desired property is measured. Three factors are important in this treatment, the initial quenching temperature (T_Q_), the stabilization quenching temperature T_S_ and the stabilization time (Δt). The typical temperature vs time profile for the stabilization quenching treatment is shown in Fig. 6b. An example performed by the group for this treatment can be seen in [11].

The main difference between the isochronal heating and the stabilization quenching treatments is that whereas the isochronal heating allows the user to measure the most stable phases formed at each temperature, the stabilization quenching allows the user to measure the phases formed from the decomposition of metastable phases.

Another possible heat treatment to study the decomposition of metastable phases is the aging treatment and the constant heating rate followed by quenching.

The aging heat treatment consists into placing the sample inside an oven with fixed temperature (T_O_) for a determined amount of time (Δt), the sample is then submitted to a fast cooling into iced water to retain the present phases in the sample. After the measurement of the desired properties, the sample is returned to the oven for an additional amount of time and the described procedure is repeated. This treatment allows the analysis of the stability and decomposition kinetics of the studied phases.

**Fig. 7:**
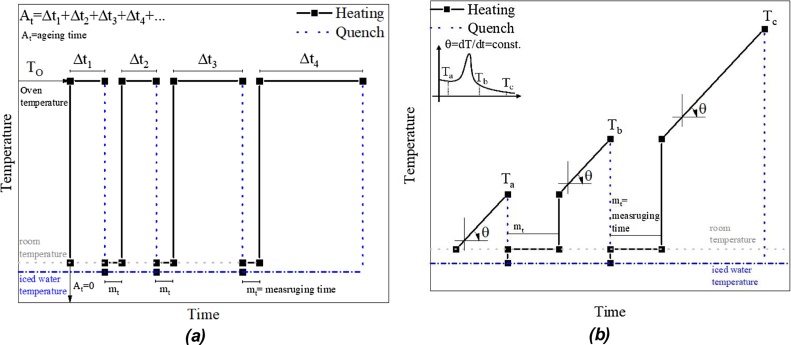
(a) Aging and (b) constant heating rate quenching.

As the aging time reaches infinity, A_t_→∞, the metastable phases transform into more stable phases until a minimum energy state is reached. To reach this limit, one important factor is the aging temperature, as the aging process is sped up in higher temperatures and slowed down at low temperatures. The temperature can also change the mechanism of the decomposition of the metastable phases, since the energy minimum reached is characteristic of the aging temperature [23]. Two factors are important in this treatment, the aging temperature (T_O_) and the aging time (A_t_). The typical temperature vs time profile for the aging is shown in Fig. 7a. An example performed by the group for this treatment can be seen in [9,15,24].

The constant heating rate quenching allows the characterization of the decomposed phases in comparison with another measurement. As an example, let us consider a DSC curve with a thermal event. If the user wants to study the phases before and after the thermal event accurately, the user can use this treatment. Placing the sample inside the oven at an initial temperature and then programming it to heat until the desired temperature at the same heating rate, and in sequence, submitting the sample to quenching the user stabilizes the stage of the decomposition at that temperature. Two factors are important in this treatment, the heating rate (θ) and the quenching temperature (T_a_). The typical temperature vs time profile for the constant heating rate is shown in Fig. 7b. An example performed by the group for this treatment can be seen in [11].

### Sample preparation

**Fig. 8:**
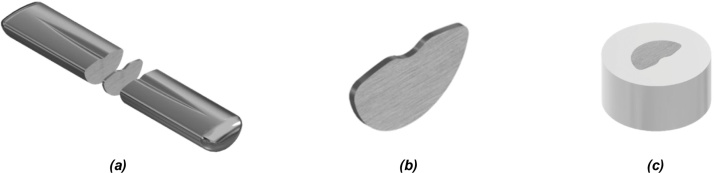
(a) Sample cut from ingot (b)sample (c) sample embedded in resin.

The sample preparation is inherent to the analysis that will be performed. Most of the sample characterization techniques require a small sample cut from the ingot. To perform this cut, a precision disc cutter can be used to minimize the heating and material loss. Usually, the sample is cut perpendicularly to the major axis of the ingot as shown in Fig. 8a. The final sample geometry is shown in Fig. 8b. After the cut procedure, the surface of the sample is much scratched from the cutting disk and further preparation is required to prepare the surface of the sample. There are two options available for surface preparation, the sample can be further prepared as it is or embedded in resin to facilitate its handling and increase the base area of the sample, as shown in Fig. 8c. As mentioned, it is necessary to eliminate any scratches and even to ensure that both sample sides are as much as possible parallel. To do so, a SiC sanding paper disk is placed onto the surface of a spinning metal disk and refrigerated constantly with water. At this configuration, the sample is then lightly pressed onto the sanding paper and sanded until the desired surface finish. To accomplish the desired surface finish, it is advised the use of different grits sandpapers, moving from the coarse grain to the finer one, in the appropriate interval. For example, if the desired surface finish is accomplished by a 1000 grit sandpaper the sample needs to be sanded at 200 grit, then 400, 600, 800 and finally the 1000 grit sandpaper can be used. In this manner, the rate of material removal is better controlled so that you can easily remove all the scratched surface from the previous sandpaper finish. If one grit is skipped the removal rate of the finer sandpaper will request the sanding more time and effort consuming.

**Fig. 9:**
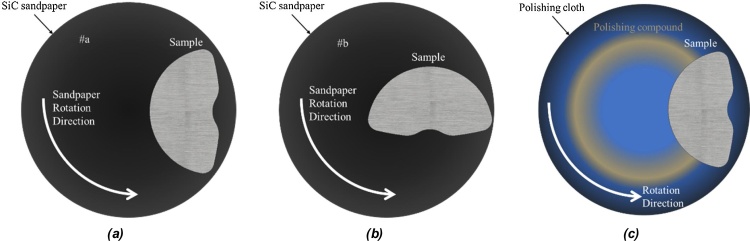
Surface preparation sanding with paper grit #a (a)and with grit #b (b), in which #a<#b and (c) polishing with cloth and compound. It is important to notice that this is a didactical illustration the sandpaper disk is 10 times bigger than the sample in real life.

To evaluate the surface finish, it is advised to place the sample in a way that the scratches in the surface are orthogonal with the tangential speed of the disk, as illustrate in Fig. 9a. When all the scratches in the sample surface get parallel to the tangential speed of the metal disk, the sandpaper can be switched to a finer one and the sample is placed orthogonally again, as illustrated in Fig. 9b. This procedure is then repeated until the desired finish is accomplished. By evaluating the surface finish in this manner, the user is assured that all the scratches promoted by the previous sandpaper are removed by the new grit. As mentioned before if coarse scratches are left in the sample surface it makes the next sanding steps way harder.

Sandpapers are available in a large grit variety reaching very high grits, such as 2000, 3000 or even high as 8000. However, the very good surface finish is reached sanding the sample until the grit 1200 and then polishing the sample with a cloth and polishing compound, considering the materials studied by the group. The type of cloth will be determined by the type of compound used; however, its placement is made analogously as the sandpaper, placing it on top of a spinning metal disk and pressing the sample against the loaded cloth, as shown in Fig. 9c.

Three types of compound are widely used to polish metal surfaces, compounds based on chromium oxides, synthetic diamond or alumina powder. The chromium oxides compounds are the cheapest ones and can last very long, however, their inherent toxicity presents an unnecessary occupational hazard as the other alternatives also lead to very good surface finishes and are much safer to use. Between alumina and synthetic diamond, the diamond polishing compound is easier to handle and does not impregnate on plastic surfaces as the alumina compounds tend to do so.

The sample polishing procedure is equal to the sanding procedure, the scratches on surface define the sample placement, changing to a finer compound when all scratches are parallel to the radial speed of the disk. However, it is important to notice that as the polishing compound gets finer the scratches onto the surface are only visible under a microscope. So, in order to check the surface finish during the polishing process, it is necessary to monitor the sample with an optical microscope to both verify the direction and size of the scratches onto the surface [25].

For correctly use the polishing compounds, a small quantity of the compound is placed onto the surface of the polishing cloth, and the correct lubricant is added to promote an easy polishing of the surface. If the compound is alumina-based, water can be used. However, if the polishing compound is based on synthetic diamond the correct lubricant is a 1:2 vol mixture of glycerin and ethanol.

After sanding the sample up to a 1200 grit the sample can be then polished using a sequence of diamond compounds with a particle size of 15, 9, 6, 3, 1 μm and then, finally, 1/4 μm. This is another advantage of the diamonds-based compounds, the availability of a great variety of particle sizes. In contrast, usually alumina suspensions can be found only with 1, 0.3 and 0.05 μm particle size. If a very fine surface finish is required, it is possible to polish the sample with diamond compound until the 1/4 μm one and then polish it with alumina. It is also important to notice that the alumina-based compounds can impregnate into the metal surface leading to detection errors of aluminum and oxygen in techniques like energy-dispersive X-ray spectroscopy.

**Fig. 10:**
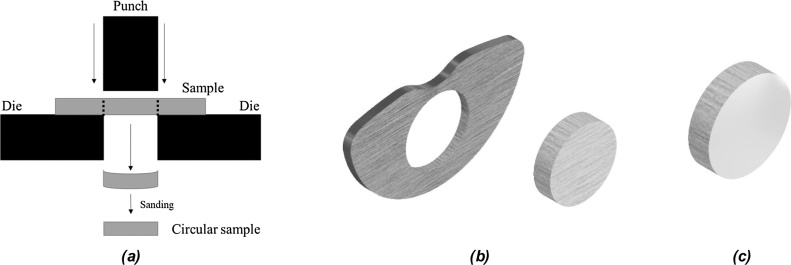
(a) punch and die setup illustration (b) punched sample and disk (c) polished sample disk.

If it is necessary that the sample should be even smaller than the described ingot slice, it is possible to punch a smaller sample from the slice. To perform this punch procedure, it is necessary to place the sample into a die, as shown in Fig. 10a. The disk sample diameter produced (as shown in Fig. 10b) can be controlled by the diameter of the punch and die set as the necessity of the analysis equipment. For instance, if it is necessary to perform a Differential Scanning Calorimetry (DSC) in a T-zero pan of TA instruments, the disk sample needs to be cut with 4 mm of diameter. However, to perform a differential thermal analysis using a Setaram alumina crucible, the sample needs to be cut with 3 mm diameter to fit inside the crucible. After the punching procedure, the disk sample surface must be prepared just like the ingot slice, producing a disk with one polished surface, as shown in Fig. 10c.

When the sample reaches the desired surface finish it is possible to etch it to selectively corrode the different phases of the sample. Many etching solutions are available in the literature [26], however, few provide detailed composition and preparation procedure and the majority focus on commercial alloys [27,28]. Table 1 shows the etching solutions used by the group, the solution composition and the alloys successfully etched by these solutions.

**Table 1:** Etching solutions composition and indicated alloys.

Etching solutions	Compositions	Alloys
Iron Chloride	17 g FeCl_3_.6H_2_O_(s)_	CuAl, CuAlMn, CuAlGd, CuAlSn, CuAlMnAg, CuAlMnBe, CuAlMnCo, CuAlMnGa, CuAlMnGd, CuAlMnSb, CuAlMnSn, CuAlMnZr, CuAlMnAgZr.
	93 ml H_2_O_(l)_	
	25 m L HCl_(aq)_ - ≈ 37%	
Nital	10 mL HNO_3(aq)_ - ≈68%	FeNiCo.
	90 mL C_2_H_5_OH_(l)_	
Chloral	2 mL HCl_(aq)_ - ≈37%	SnCu, SnCuAg.
	98 mL C_2_H_5_OH_(l)_	
Aqua regia	10 mL HCl_(aq)_ - ≈37%	FeCuNiMnTiSn, FeNiCuMnTiSnAlV.
	10 mL HNO_3(aq)_ - ≈ 68%	
Ammonium Persulfate I	40 g (NH_4_)S_2_O_8(s)_	CuAlMnAg.
	75 mL H_2_O_(l)_	
	50 mL H_2_O_2(l)_	
Ammonium Persulfate II	10 g (NH_4_)S_2_O_8(s)_	CuAl, CuAlAg, CuAlBe, CuAlCo, CuAlGa, CuAlGd, CuAlNi, CuAlZr.
	100 m L H_2_O_(l)_	

To proceed the etch the sample is cleaned with acetone to de-grease the surface and then the etching solution is dropped onto the sample surface using a pipette. As time passes the sample is submitted to a controlled corrosion process, the intensity of this procedure is controlled by managing the time that the solution stays in contact with the sample. Ideally, the process is carried until a good contrast is observed between the phases in the alloy. After the desired time, the solution is washed off the sample surface with water or ethanol, and the sample is dried using a compressed air pistol. The time can be controlled in a systematic way, where the sample is etched by a very short time interval (such as 1 s) and then examined by optical microscope, checking if the desired contrast was achieved, if not, the sample is submitted to a new etching procedure.

In Table 1 the alloys with published results are: CuAl [[1], [2], [3], [4], [5]]; SnCu [6]; CuAlZr [4]; CuAlCo [4]; CuAlSn [5]; Cu AlGd [2,3,5]; CuAlMn [[1], [2], [3],^5^,[7], [8], [9], [10]]; SnCuAg [6]; CuAlAg [1]; FeNiCo [11]; CuAlMnAg [1,[7], [8], [9], [10],[12], [13], [14], [15]]; CuAlMnSn [5]; CuAlMnGd [2,3,5]. All other alloys are work in progress and not published, but the research group already used the method presented in this paper obtaining good results.

It is important to notice that if the solution is let to react too much time with the sample its surface will get over etched, in other words, the corrosion process will promote inappropriate contrast between the phases. If this is the case, the sample needs to be sanded again to remove the corroded surface and re-polished to only then be re-etched. If the etching process however only got a little bit too aggressive, re-polishing the surface of the sample will soften up the contrast between the phases and can produce a suitable sample. However, this process can contaminate the polishing cloth with an etching solution and should be avoided.

Some important care and precautions need to be noted to prepare the etching solutions. For preparing the ammonium persulfate solution it is advised to prepare a 50 mL saturated solution at room temperature and then complete with water until the final volume of 75 mL and leave the addition of hydrogen peroxide as final procedure. To prepare the iron chloride solution it is advised to prepare an acidic solution with the concentrated acid and water first and then dissolves the iron chloride in it, as the solid will dissolve much faster in this manner. To prepare the Nital solution it is advised to work with cold ethanol and maintain the solution refrigerated to avoid the formation of ethyl nitrate and maintain the final solution in a closed, but not hermetic, container. It is also advised to not surpass 10% concentration, if this is needed it is recommended using methanol instead of ethanol, keeping in mind that methanol has low acute toxicity and the daily contact with this substance needs to be minimal (002 g of methanol by m³ of air) [29]. All solutions can be extensively reused and its waste recycled into its original form. All solutions can be made using distilled or ultrapure water, tap water is not recommended.

It is always advised to handle chemicals in a fume hood using the correct personal protective equipment and dispose correctly of the chemical waste generated during the etching process. It is important to notice that Nital, *aqua regia* and the ammonium persulfate solutions spontaneously produce gases at room temperature, and should never be stored in hermetic containers when handled at room temperature. This gases are toxic or inflammable and are produced during the decomposition of the chemical compounds responsible for the etching, reducing the solution effectiveness and increasing the vessel pressure. Caution is advised when storing these solutions. To increase the lifetime of the prepared solution and decrease its vapors emissions it is advised to keep all the solutions of Table 1 under refrigeration, preferably, in a freezer that reaches -18 °C or lower. At this point the decomposition of the etching solutions is negligible, and as long as the solutions are kept in this temperature and not cycled to ambient temperature and back, they can be used for long periods of time before losing effectiveness.

To use the stored solutions the correct procedure is to remove an aliquot of the refrigerated solution into a non-sealed flask and then, under the fume hood, let the solution equilibrate at ambient temperature. After equilibrating the temperature is recommended to use this solution to etch a calibration sample, one that the user knows very well its structure and reaction time to test the effectiveness of the etching solution. If the calibration sample presents the desired contrasts levels then the solution can be used to etch new samples. It is important to notice that the temperature of the solution will affect the etching time since it will affect the corrosion rate in a manner that warmer solutions will etch faster than cold solutions. To contour this problem, it is recommended to perform the etching in an ambient with controlled temperature to keep consistency between samples. Finally, it is important to notice that controlling the temperature may lead to low air humidity which can also impact the etching process, so it is recommended to etch samples in an ambient with humidity between 50% and 90%.

**Fig. 11:**
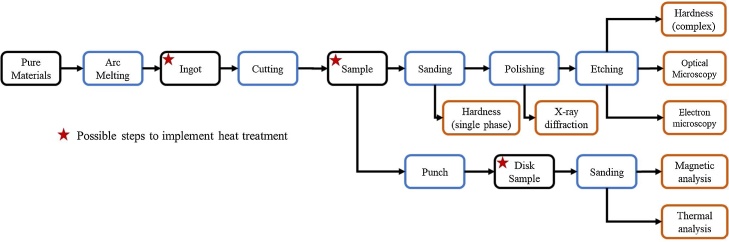
Sample preparation requirements for different experimental techniques.

To characterize the prepared alloy, it is necessary to prepare a sample accordingly to the characterization method requirements. For instance, to perform optical microscopy it is necessary to sand, polish and etches the sample, however, to perform thermal analysis it is not necessary neither to polish or etch the sample. Fig. 11 shows the necessary procedure for each characterization employed for phase transformation analysis. It is important to notice that thermal treatments can be inserted before different steps, depending on the study objectives. It is also important to note that for electrons microscopy extra steps may be needed. For Scanning Electron Microscopy (SEM) the polishing compound need to be thoroughly cleaned from the surface and even than alumina-based compounds can jeopardize measurements such as energy-dispersive spectroscopy or backscattered images. And even further than that, to prepare samples for Transmission Electron Microscopy (TEM) it is necessary to taper down the sample until it presents a region with microns or even nanometers thickness. These proceeding will be dependent of the sample type and probably will be carried out by an trained technician following available methods for each type of sample available in the literature for TEM [27,[30], [31], [32], [33]] and SEM [[34], [35], [36]].
